# Mucosal-Associated Invariant T Cells Accumulate in the Lungs during Murine *Pneumocystis* Infection but Are Not Required for Clearance

**DOI:** 10.3390/jof8060645

**Published:** 2022-06-18

**Authors:** Lisa R. Bishop, Shelly J. Curran, Joseph A. Kovacs

**Affiliations:** Critical Care Medicine Department, NIH Clinical Center, National Institutes of Health, Bethesda, MD 20892, USA; lbishop@cc.nih.gov (L.R.B.); shelly.curran@nih.gov (S.J.C.)

**Keywords:** *Pneumocystis*, MAIT cells, mouse models, pneumonia

## Abstract

*Pneumocystis* is a fungal pathogen that can cause pneumonia in immunosuppressed hosts and subclinical infection in immunocompetent hosts. Mucosal-associated invariant T (MAIT) cells are unconventional lymphocytes with a semi-invariant T-cell receptor that are activated by riboflavin metabolites that are presented by the MHC-1b molecule MR1. Although *Pneumocystis* can presumably synthesize riboflavin metabolites based on whole-genome studies, the role of MAIT cells in controlling *Pneumocystis* infection is unknown. We used a co-housing mouse model of *Pneumocystis* infection, combined with flow cytometry and qPCR, to characterize the response of MAIT cells to infection in C57BL/6 mice, and, using MR1^−/−^ mice, which lack MAIT cells, to examine their role in clearing the infection. MAIT cells accumulated in the lungs of C57BL/6 mice during *Pneumocystis* infection and remained at increased levels for many weeks after clearance of infection. In MR1^−/−^ mice, *Pneumocystis* infection was cleared with kinetics similar to C57BL/6 mice. Thus, MAIT cells are not necessary for control of *Pneumocystis* infection, but the prolonged retention of these cells in the lungs following clearance of infection may allow a more rapid future response to other pathogens.

## 1. Introduction

*Pneumocystis* is a fungus that can cause inapparent pulmonary infection in immunocompetent hosts but can cause potentially life-threatening pneumonia (PCP) in immunocompromised hosts [1]. Patients with HIV infection, as well as those with malignancies, transplants, and autoimmune diseases, are at increased risk of developing PCP [2]. Corticosteroids are a well-defined risk factor for developing PCP in these latter settings, but other drugs, including newer biologics, can also increase this risk. 

An improved understanding of host immune responses to *Pneumocystis* should provide insights into the immune defects that allow PCP to develop and may help to better identify patients at the highest risk of developing PCP. Mucosal-associated invariant T (MAIT) cells are innate T cells that are highly conserved throughout mammalian evolution [3]. MAIT cells are alpha beta T cells that have semi-invariant T-cell receptors, which recognize riboflavin metabolites from pathogens and commensals when presented by major histocompatibility-related molecule 1 (MR1). Of note, MAIT cells develop early in life upon exposure to riboflavin metabolites [4]. Other MR1 ligands include folic acid derivatives and several drug and drug-like molecules [5]. MAIT cells have been associated with several disease categories [6], including infectious diseases, such as HIV/AIDS [7,8], sepsis, and tuberculosis, as well as asthma, type 2 diabetes, and obesity [9]. Following activation, MAIT cells develop effector functions, including secretion of cytokines, primarily IL-17A in mice [10], as well as cytotoxicity [11]. MAIT cells have also been shown to promote tissue repair [4,12]. MAIT cells are increasingly being considered for use in immunotherapy for various cancers and for use in vaccine development [13,14,15,16]. 

The availability of high-quality *Pneumocystis* genomes allows us to explore not only the biology of *Pneumocystis* but also its potential interactions with host immune defenses. The set of genes needed for riboflavin biosynthesis have been identified in all *Pneumocystis* species except possibly *Pneumocystis canis* (which appears to be missing a single gene) [17,18], suggesting that MAIT cells could play a role in controlling *Pneumocystis* infection. To explore this hypothesis, we utilized mouse models of *Pneumocystis* infection to examine the kinetics of MAIT cells in the lung and whether their absence impacts infection.

## 2. Materials and Methods

### 2.1. Animals

C57BL/6 mice were obtained from the National Institutes of Health (NIH, Bethesda, MD) or The Jackson Laboratory (Bar Harbor, ME). CD40 ligand knockouts (CD40L-KO, strain B6, 129S-Tnfsf5^tm1lmx^/J), which are highly susceptible to *Pneumocystis* infection, were obtained from The Jackson Laboratory. MR1^−/−^ mice (on a C57BL/6 background), which lack MAIT cells, were generated by Dr. Susan Gilfillan and colleagues [19] and were kindly provided by Drs. Michael Constantinides and Yasmine Belkaid; their genotype was verified by PCR [19]. Mice were subsequently housed in microisolator cages in ventilated racks and bred at the NIH Clinical Center animal facilities. All animal studies were performed under an NIH Clinical Center Animal Care and Use Committee-approved protocol. 

### 2.2. P. murina Infection Model

Female mice, typically 6–8 weeks old, were infected by co-housing with female *P. murina* infected seeder mice (CD40L-KO) and sacrificed at various time points [20,21]. This model more closely resembles natural infection via inhalation of organisms than an intratracheal inoculation model and provides predictable kinetics, with a peak of infection in immunocompetent mice at ~5 to 6 weeks and clearance by ~9 to 11 weeks post initial exposure. Female mice were utilized because co-housed non-litter males are combative. Unexposed litter mate controls (male and female) were housed in clean facilities; to minimize the number of animals used per experiment, 1–2 unexposed mice per time point were sacrificed and processed simultaneously with exposed mice, and data from these unexposed mice were combined as controls. 

To compare infection and immune responses, different strains of mice were co-housed to maintain identical exposure conditions. Blood, lungs, and, in 1 experiment, spleens, from 2 to 4 animals per strain per time point, were collected. Serum was analyzed for anti-*P. murina* antibodies by ELISA; a portion of the lungs was analyzed for *P. murina* infection by qPCR; and lung and spleen were processed for flow cytometry.

### 2.3. Quantitation of P. murina by qPCR

To quantitate *P. murina* organism burden, DNA was extracted from a piece of lung using the QIAamp DNA Mini Kit (Qiagen, Germantown, MD, USA), followed by qPCR targeting the *P. murina* dihydrofolate reductase gene (*dhfr*), a single-copy gene, using the ViiA 7 Real-Time PCR System (Applied Biosystems, Waltham, MA, USA) or CFX384 Touch Real-Time PCR Detection System (Bio-Rad, Hercules, CA, USA), as previously described [20,22]. Results are expressed as log_10_ *dhfr* copies/mg lung tissue.

### 2.4. ELISA

Anti-*P. murina* antibodies were measured by ELISA using a crude *P. murina* lysate as previously described [23]. Briefly, *Pneumocystis* organisms were partially purified by Ficoll-Hypaque density gradient centrifugation and disrupted with glass beads, followed by sonication and centrifugation; the resulting supernatant was utilized to coat the wells. Wells were incubated with mouse serum (1:100 dilution), followed by secondary antibody (1:1000 dilution; Peroxidase AffiniPure Goat Anti-Mouse IgG (H+L) (Jackson ImmunoResearch Laboratories Inc., West Grove, PA, USA), and developed using OPD substrate (Sigma-Aldrich, Inc., St. Louis, MO, USA). 

### 2.5. Flow Cytometry

For single-cell suspensions, lung and spleen tissues were digested with Lung Dissociation Kit, mouse and Spleen Dissociation Kit, mouse (Miltenyi Biotec, Bergisch Gladbach, Germany), respectively, using the gentleMACS Octo Dissociator with Heaters (Miltenyi Biotec), per manufacturer’s recommendations.

The following antibodies were used for immunophenotyping: CD19 PE-Vio^®^770 (clone 6D5), CD4 APC-Vio^®^770 (GK1.5), (Miltenyi Biotec) CD3e FITC (145-2C11), CD19 PE-Cy^TM^7 (1D3), CD4 APC-H7 (GK1.5), CD8a BV510 (53-6.7), TCR β chain PerCP-Cy^TM^5.5 (H57-597), CD11b PE-Cy^TM^7 or APC (M1/70), IFNγ APC (XMG1.2), IL-4 APC (11B11), IL-5 APC (TRFK5) (BD Biosciences, Franklin Lakes, NJ, USA) and IL-17 APC (TC11-18H10) (BioLegend, San Diego, CA, USA). MR1 5-(2-oxopropylideneamino)-6-D-ribitylaminouracil (5-OP-RU) PE-labeled tetramers and MR1 6-formyl pterin (6-FP; control) PE-labeled tetramers were provided by the NIH Tetramer Facility [24]. 

**Figure 1:**
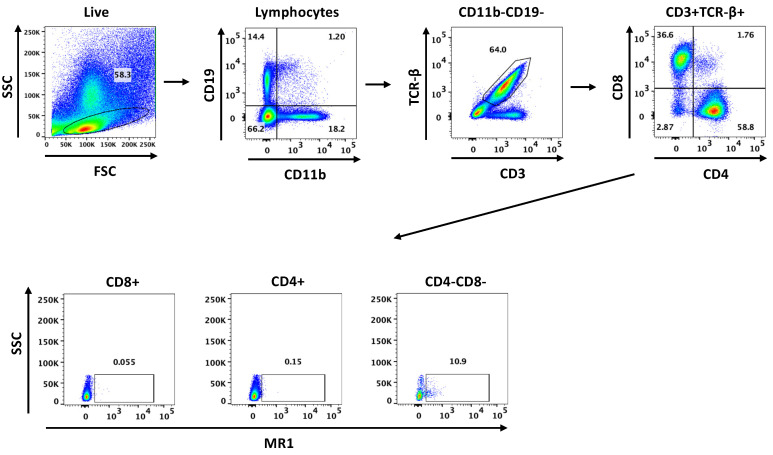
Flow cytometry gating strategy for the detection of MAIT cells in the lungs of C57BL/6 mice. CD19^+^ and CD11b^+^ cells were excluded from live lymphocytes singlets. CD3^+^TCR^−^β^+^ T cells were then gated on CD11b^−^CD19^−^ cells followed by CD4 and CD8. To identify MAIT cells, CD8^+^, CD4^+^ and CD4^−^CD8^−^ (DN) T cells were analyzed for binding of 5-OP-RU MR1 tetramer. 5-FP MR1 control tetramer was used as a negative control to gate MAIT cells, which were primarily found in the DN T-cell population.

For flow cytometry analysis, 1–2 million cells per tube were labeled with Live/Dead Fixable Violet Dead Cell Stain (Molecular Probes, Eugene, OR, USA), Fc receptors were blocked, and cells were subsequently labeled with antibodies plus tetramer for 1 h at room temperature and then fixed with fixation buffer (BD Biosciences). Data were acquired on a MACSQuant 10 analyzer or Fortessa LSR Flow Cytometer and analyzed using FlowJo Software (version 10.8.1; BD Life Sciences, Ashland, OR, USA). MAIT cells were present almost exclusively in the CD3^+^TCRβ^+^CD4^−^CD8^−^ (double negative (DN)) T-cell populations and are thus reported as a percent of DN T cells. Figure 1 illustrates the gating strategy for identifying MAIT cells.

### 2.6. Statistics

Cell populations at individual time points were compared to baseline values using unpaired, two-sided Student’s *t*-test, using Prism (version 9, GraphPad Software, LLC, San Diego, CA, USA) or Excel (version 16.59, Microsoft, Redmond, WA, USA). 

## 3. Results

### 3.1. MAIT Cells Accumulate in the Lungs of C57BL/6 Mice during P. murina Infection

**Figure 2:**
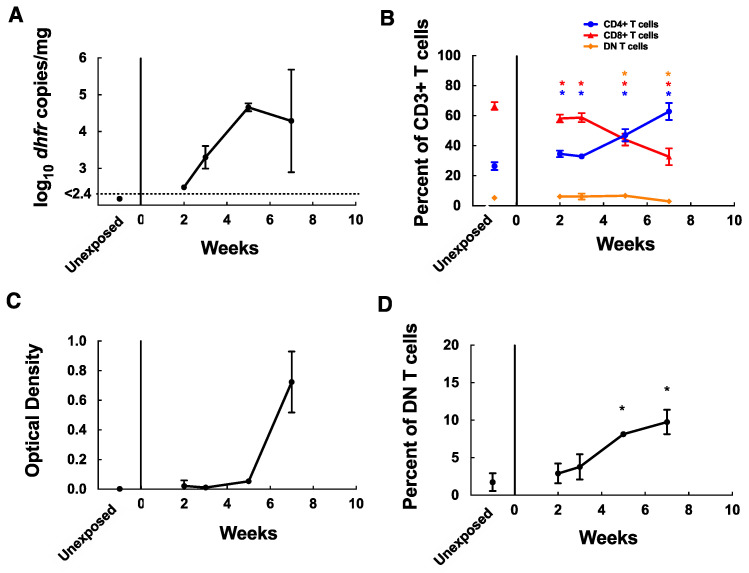
MAIT cells accumulate in the lungs of *P. murina* infected C57BL/6 mice. (**A**) Quantitation by qPCR of *Pneumocystis* organism load in the lungs of C57BL/6 mice exposed to *P. murina* infected seeder mice for 2, 3, 5, and 7 weeks and in unexposed controls. Results are shown as log_10_ *dhfr* copies/mg lung tissue. The dotted line represents the lower limit of detection of the assay. (**B**) Flow cytometry characterization of T-cell populations in the lungs of C57BL/6 mice during *P. murina* infection. CD4^+^ T cells, CD8^+^ T cells, and CD4^−^CD8^−^ (DN) T cells are shown as percent of CD3^+^ T cells. (**C**) ELISA results (presented as the optical density 450 nm) measuring anti-*P. murina* serum antibodies using a crude antigen preparation of partially purified *P. murina* organisms. (**D**) Flow cytometry results demonstrating MAIT cells detected in the lungs of C57BL/6 mice using 5-OP-RU loaded MR1 tetramer, shown as percent of CD4^−^CD8^−^ (DN) T cells. Data points represent the mean value at each time point, error bars represent standard deviations and *p*-values < 0.05 when compared to baseline values in panels (**B**,**D**) are indicated by *. For panel (**B**) the color of the asterisks indicates which cell population it refers to. For all panels, for exposed mice *n* = 3 each for weeks 2, 3, and 5, and *n* = 2 for week 7, and for unexposed controls, *n* = 4.

Given the potential role of MAIT cells in immunity to bacterial and fungal pathogens, we undertook studies to examine their role in mouse models of *Pneumocystis* infection. To determine whether acute *Pneumocystis* infection induces accumulation of MAIT cells in the lungs of immunocompetent mice, C57BL/6 mice were euthanized at 2, 3, 5, and 7 weeks following the start of co-housing with an infected seeder, and lungs and spleens were harvested. Low numbers of *P. murina* organisms were detected by qPCR in the lungs at 2 weeks (mean 310 copies/mg tissue), with a gradual increase over time (Figure 2A). As previously reported [25], CD4^+^ T cells as a percent of CD3^+^ T cells increased in the lungs during infection, while CD8^+^ T cells decreased (Figure 2B), and anti-*Pneumocystis* antibodies developed by week 7 (Figure 2C). MAIT cells (MR1 5OP-RU tetramer positive) were identified almost exclusively in the DN T-cell population, which accounted for <10% of T cells (Figure 1 and Figure 2B). MAIT cells were present at low frequencies in the lungs of unexposed C57BL/6 mice (~1.7% of DN T cells) but accumulated in the lungs of *Pneumocystis*-infected mice after 2 weeks (mean, 2.9% of DN T cells), peaking at 7 weeks’ (9.7% DN T cells) exposure (Figure 2D). The trends were similar when MAIT cells were characterized as a percent of total CD3^+^ T cells rather than DN T cells. During this same time frame, MAIT cells accounted for <1.0% of DN T cells in the spleen through week 5, with a small increase to 1.3% at week 7 (data not shown). 

### 3.2. MAIT Cells Are Not Required for Control of P. murina Infection

**Figure 3:**
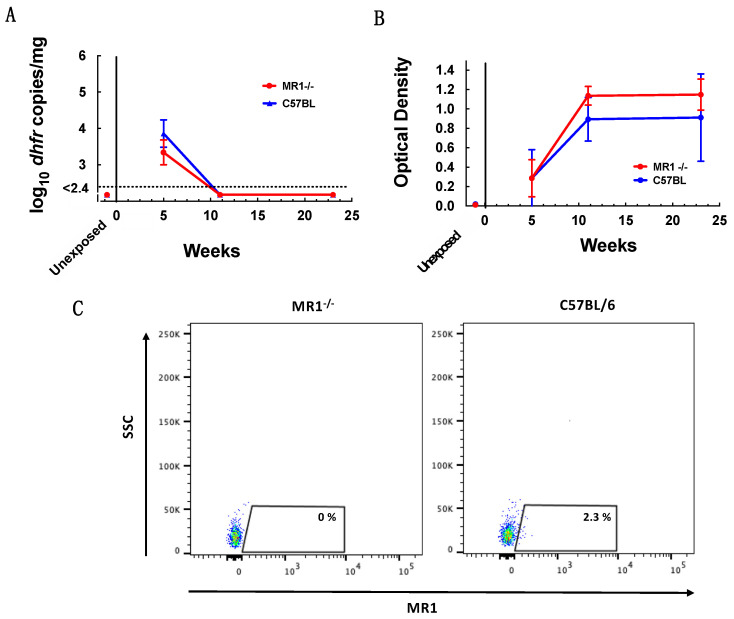
The absence of MAIT cells does not delay or prevent clearance of *P. murina* infection. (**A**) Quantitation by qPCR of *Pneumocystis* organism load in the lungs of MR1^−/−^ mice and C57BL/6 mice following exposure to *P. murina* infected seeder mice for 5, 11, and 23 weeks. Unexposed MR1^−/−^ and C57BL/6 control mice were uninfected. Results are shown as log_10_ *dhfr* copies/mg lung tissue. The dotted line represents the lower limit of detection of the assay. (**B**) ELISA results (presented as optical density 450 nm) measuring anti-*P. murina* serum antibody levels. (**C**) Representative flow cytometry plots analyzing binding of 5-OP-RU MR1 tetramers to identify MAIT cells from day 35 for MR1^−/−^ (**left**) and C57BL/6 (**right**) mice; gating was on DN T cells, as described in Figure 1. For (**A**,**B**), data points represent the mean value at each time point, and error bars represent standard deviations. MR1^−/−^ and C57BL/6 mice showed no significant difference in organism burden, kinetics/clearance of infection or development of anti-*Pneumocystis* antibodies. For both panels, for exposed mice *n* = 3 each for weeks 5 and 11, and *n* = 4 for week 23 for each strain, and for unexposed controls, *n* = 3 for MR1^−/−^ and *n* = 6 for C57BL/6 mice. All mice were genotyped to confirm they were the correct strain.

Since MAIT cells accumulated in the lung of C57BL/6 mice during *Pneumocystis* infection, we next sought to determine whether MAIT cells impact clearance of infection and whether the absence of MAIT cells would affect anti-*P. murina* antibody responses. To address this question, we co-housed MR1^−/−^ mice, which lack MAIT cells, and C57BL/6 mice with *P. murina* infected seeder mice. We found that after 5 weeks, MR1^−/−^ mice had similar *P. murina* organism loads as C57BL/6 mice and that both groups had cleared infection by week 11, demonstrating that MAIT cells are not required to control or clear *P. murina* infection (Figure 3A). Similar levels of anti-*P. murina* antibodies were present in serum by week 5 in both MR1^−/−^ and C57BL/6 mice (mean O.D. 0.29 for both) with increases by week 11 (mean O.D. MR1 KO 1.14 and WT 0.90) (Figure 3B). MAIT cells were detected by flow cytometry at week 5 in C57BL/6 mice but not in MR1^−/−^ mice, verifying the phenotype of the latter strain (Figure 3C).

### 3.3. MAIT Cells Remain Elevated in Lungs after Clearance of P. murina Infection

**Figure 4:**
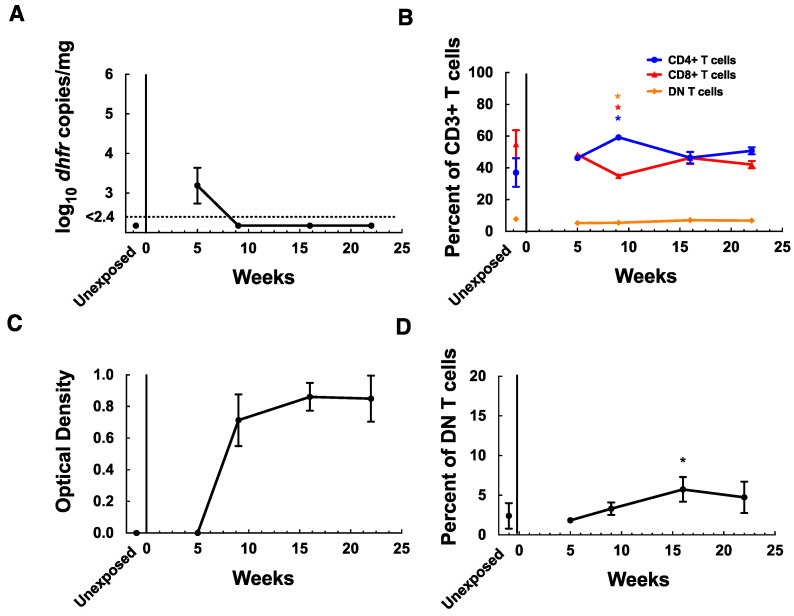
MAIT cells are retained in the lungs of C57BL/6 mice following clearance of *P. murina* infection. (**A**) Quantitation by qPCR of *Pneumocystis* organism load in C57BL/6 mice after 5, 9, 16, and 22 weeks’ exposure to *P. murina* infected seeder mice, and in unexposed controls. Results are shown as log_10_ *dhfr* copies/mg lung tissue. The dotted line represents the lower limit of detection of the assay. (**B**) Flow cytometry results demonstrating T-cell populations in the lungs of C57BL/6 mice infected with *P. murina*, including CD4^+^ T cells, CD8^+^ T cells, and CD4^−^CD8^−^ (DN) T cells, shown as a percent of CD3^+^ T cells. (**C**) ELISA results (presented as the optical density 450 nm) measuring anti-*P. murina* serum antibodies. (**D**) Flow cytometry results demonstrating MAIT cells detected in the lungs of C57BL/6 mice using 5-OP-RU loaded MR1 tetramer, shown as a percent of CD4^−^CD8^−^ (DN) T cells. Data points represent the mean value at each time point, error bars represent standard deviations, and *p*-values < 0.05 when compared to baseline values in panels (**B**,**D**) are indicated by *. For panel (**B**) the color of the asterisks indicates which cell population it refers to. For all panels, for exposed mice *n* = 2 for week 5, and *n* = 3 each for weeks 9, 16, and 22, and for unexposed controls, *n* = 4.

Given that MAIT cells may play a role in protecting against other respiratory pathogens, including bacteria and viruses, such as influenza, we wanted to determine whether MAIT cells remained in the lungs of mice for a prolonged period of time following clearance of *P. murina* infection. Immunocompetent C57BL/6 mice typically clear infection by week 9–11 post-exposure. To determine whether MAIT cells were still increased in the lungs following clearance, we looked at MAIT cells in the lungs of C57BL/6 mice for up to 22 weeks after initial exposure. C57BL/6 mice were infected by week 5 (mean 1986 copies/mg tissue) and had cleared infection by week 9, as expected; changes in lymphocyte populations and anti-*P. murina* antibodies were consistent with prior studies (Figure 4A–C) [25]. MAIT cells were elevated above controls from weeks 9 to 22, though only statistically significantly elevated at week 16 (5.7% of DN T cells, Figure 4D). When expressed as a percent of CD3+ T cells, MAIT cells were above control values at weeks 16 and 22, though neither was statistically significant. Similarly, in mice that were exposed to a seeder for only 1 week rather than continuously, MAIT cells accounted for 6.3% of DN T cells at week 20.

## 4. Discussion

In the current study, we showed that MAIT cells, which recognize riboflavin metabolites, accumulate in the lungs of immunocompetent C57BL/6 mice during *Pneumocystis* infection, but the absence of MAIT cells does not delay or prevent clearance. Moreover, MAIT cells are retained in the lungs following *Pneumocystis* infection well beyond the time that organisms can be detected. 

MAIT cells require riboflavin (vitamin B2) metabolites for development and are primarily activated when these metabolites are presented to their semi-invariant T-cell receptor by MR1, an MHC class 1b molecule; they can also be activated through TLRs and directly by cytokines [3,4]. The influx of MAIT cells into the lungs of immunocompetent mice infected with *Pneumocystis* provides support for the inference from genome sequencing studies that most *Pneumocystis* species have the necessary enzymes to synthesize riboflavin [17,18], although it remains possible that they are activated by an alternative mechanism, as noted above. 

While *Pneumocystis* infection leads to an accumulation of MAIT cells in the lung, the similar kinetics of infection and antibody responses in C57BL/6 and MR1^−/−^ mice clearly demonstrate that MAIT cells are not needed for control of infection. However, it is possible that MAIT cells play a minor role in altering the kinetics of infection; we did not conduct more detailed kinetics, as our primary goal was to determine if *Pneumocystis* infection could be cleared in the absence of MAIT cells. 

In mice, activated MAIT cells primarily produce IL-17A, a cytokine important for control of some fungal infections, but not Pneumocystis, as IL-17A knockout mice can control the infection similarly to C57BL/6 mice [26]. We attempted to characterize the cytokine profile of lung MAIT cells during infection. While IL-17 seemed to be the main cytokine produced, the loss of a high proportion of MAIT cells following in vitro activation, possibly due to MAIT cell death or downregulation of the T-cell receptor, did not allow firm conclusions in this regard. 

We also found that CD40 KO and CD40L KO mice, both of which are highly susceptible to *Pneumocystis* infection, had measurable levels of MAIT cells similar to those found in C57BL/6 mice (data not shown), suggesting that MAIT cells are insufficient to control infection in the setting of a defective adaptive immune response. 

MAIT cells were retained in the lungs for many weeks following clearance of *Pneumocystis*, similar to what has been reported for other infections, such as *Salmonella typhimurium* [10], where MAIT cells accumulated rapidly following intranasal inoculation and remained elevated through 7 weeks. Since humans and other host species are exposed to *Pneumocystis* at a very young age, this retention of MAIT cells following infection may provide a benefit to the host, as MAIT cells would be on site to respond more rapidly to other infections in which they may play a more important role. 

It is important to note that MAIT cells are much more abundant in humans than mice [27], and thus, the conclusions of this study may not be applicable to humans. In transgenic models with larger numbers of MAIT cells, the importance of these cells in controlling infection with, e.g., *E. coli,* has been easier to demonstrate [28]. However, MAIT cells are much less numerous in infants under 2 years of age compared to older humans [27], and nearly all humans appear to be infected with *Pneumocystis* prior to age 1 [29], suggesting that at least for primary infection, the results may be applicable to humans. 

We did not have lung weights available and thus could not express changes in MAIT cells per mg lung tissue; it is possible that these changes represent relative and not absolute increases in lung MAIT cells. However, we noted an increase in the percent of CD3^+^ T cells in co-housed mice compared to controls (data not shown), and we have previously shown by immunohistochemistry that there was an increase in lymphocytes, primarily CD4^+^ T cells and B cells, as well as macrophages, in C57BL/6 mice infected with *Pneumocystis* [25]. When MAIT cells were expressed as a percent of CD3^+^ T cells, similar trends were seen as when they were expressed as a percent of DN T cells. 

In summary, MAIT cells accumulate in the lungs in response to murine *Pneumocystis* infection but do not appear to have a role in controlling or clearing infection. MAIT cells are retained in the lungs for many weeks after infection, which may have a beneficial role in controlling future pulmonary infections.

## Data Availability

The data presented in this study are available within the article.

## References

[1] Kovacs J.A., Masur H. (2009). Evolving health effects of *Pneumocystis*: One hundred years of progress in diagnosis and treatment. JAMA.

[2] Carmona E.M., Limper A.H. (2011). Update on the diagnosis and treatment of *Pneumocystis* pneumonia. Ther. Adv. Respir. Dis..

[3] Godfrey D.I., Koay H.F., McCluskey J., Gherardin N.A. (2019). The biology and functional importance of MAIT cells. Nat. Immunol..

[4] Constantinides M.G., Link V.M., Tamoutounour S., Wong A.C., Perez-Chaparro P.J., Han S.-J., Chen Y.E., Li K., Farhat S., Weckel A. (2019). MAIT cells are imprinted by the microbiota in early life and promote tissue repair. Science.

[5] Keller A.N., Eckle S.B., Xu W., Liu L., Hughes V.A., Mak J.Y., Meehan B.S., Pediongco T., Birkinshaw R.W., Chen Z. (2017). Drugs and drug-like molecules can modulate the function of mucosal-associated invariant T cells. Nat. Immunol..

[6] Sugimoto C., Fujita H., Wakao H. (2016). Mucosal-associated invariant T cells from induced pluripotent stem cells: A novel approach for modeling human diseases. World J. Stem Cells.

[7] Cosgrove C., Ussher J.E., Rauch A., Gartner K., Kurioka A., Huhn M.H., Adelmann K., Kang Y.-H., Fergusson J.R., Simmonds P. (2013). Early and nonreversible decrease of CD161^++^/MAIT cells in HIV infection. Blood.

[8] Leeansyah E., Ganesh A., Quigley M.F., Sonnerborg A., Andersson J., Hunt P.W., Somsouk M., Deeks S.G., Martin J.N., Moll M. (2013). Activation, exhaustion, and persistent decline of the antimicrobial MR1-restricted MAIT-cell population in chronic HIV-1 infection. Blood.

[9] Magalhaes I., Pingris K., Poitou C., Bessoles S., Venteclef N., Kiaf B., Beaudoin L., Da Silva J., Allatif O., Rossjohn J. (2015). Mucosal-associated invariant T cell alterations in obese and type 2 diabetic patients. J. Clin. Investig..

[10] Chen Z., Wang H., D’Souza C., Sun S., Kostenko L., Eckle S.B., Meehan B.S., Jackson D.C., Strugnell R.A., Cao H. (2017). Mucosal-associated invariant T-cell activation and accumulation after in vivo infection depends on microbial riboflavin synthesis and co-stimulatory signals. Mucosal Immunol..

[11] Leeansyah E., Svard J., Dias J., Buggert M., Nystrom J., Quigley M.F., Moll M., Sonnerborg A., Nowak P., Sandberg J.K. (2015). Arming of MAIT Cell Cytolytic Antimicrobial Activity Is Induced by IL-7 and Defective in HIV-1 Infection. PLoS Pathog..

[12] Leng T., Akther H.D., Hackstein C.P., Powell K., King T., Friedrich M., Christoforidou Z., McCuaig S., Neyazi M., Arancibia-Carcamo C.V. (2019). TCR and Inflammatory Signals Tune Human MAIT Cells to Exert Specific Tissue Repair and Effector Functions. Cell Rep..

[13] Hinks T.S.C. (2021). Boosting MAIT cells as immunotherapy: Context is everything. Mucosal Immunol..

[14] Wang H., Kjer-Nielsen L., Shi M., D’Souza C., Pediongco T.J., Cao H., Kostenko L., Lim X.Y., Eckle S.B.G., Meehan B.S. (2019). IL-23 costimulates antigen-specific MAIT cell activation and enables vaccination against bacterial infection. Sci. Immunol..

[15] Bohineust A., Tourret M., Derivry L., Caillat-Zucman S. (2021). Mucosal-associated invariant T (MAIT) cells, a new source of universal immune cells for chimeric antigen receptor (CAR)-cell therapy. Bull. Cancer.

[16] Masina N., Bekiswa A., Shey M. (2021). Mucosal-associated invariant T cells in natural immunity and vaccination against infectious diseases in humans. Curr. Opin. Immunol..

[17] Ma L., Chen Z., Huang D.W., Kutty G., Ishihara M., Wang H., Abouelleil A., Bishop L., Davey E., Deng R. (2016). Genome analysis of three *Pneumocystis* species reveals adaptation mechanisms to life exclusively in mammalian hosts. Nat. Commun..

[18] Cisse O.H., Ma L., Dekker J.P., Khil P.P., Youn J.-H., Brenchley J.M., Blair R., Pahar B., Chabe M., Van Rompay K.K.A. (2021). Genomic insights into the host specific adaptation of the *Pneumocystis* genus. Commun. Biol..

[19] Treiner E., Duban L., Bahram S., Radosavljevic M., Wanner V., Tilloy F., Affaticati P., Gilfillan S., Lantz O. (2003). Selection of evolutionarily conserved mucosal-associated invariant T cells by MR1. Nature.

[20] Vestereng V.H., Bishop L.R., Hernandez B., Kutty G., Larsen H.H., Kovacs J.A. (2004). Quantitative real-time polymerase chain-reaction assay allows characterization of *Pneumocystis* infection in immunocompetent mice. J. Infect. Dis..

[21] Bishop L.R., Lionakis M.S., Sassi M., Murphy P.M., Hu X., Huang D.W., Sherman B., Qiu J., Yang J., Lempicki R.A. (2015). Characterization of chemokine and chemokine receptor expression during *Pneumocystis* infection in healthy and immunodeficient mice. Microbes Infect..

[22] Liu Y., Davis A.S., Ma L., Bishop L., Cisse O.H., Kutty G., Kovacs J.A. (2020). MUC1 mediates *Pneumocystis* murina binding to airway epithelial cells. Cell. Microbiol..

[23] Bishop L.R., Helman D., Kovacs J.A. (2012). Discordant antibody and cellular responses to *Pneumocystis* major surface glycoprotein variants in mice. BMC Immunol..

[24] Corbett A.J., Eckle S.B., Birkinshaw R.W., Liu L., Patel O., Mahony J., Chen Z., Reantragoon R., Meehan B., Cao H. (2014). T-cell activation by transitory neo-antigens derived from distinct microbial pathways. Nature.

[25] Hernandez-Novoa B., Bishop L., Logun C., Munson P.J., Elnekave E., Rangel Z.G., Barb J., Danner R.L., Kovacs J.A. (2008). Immune responses to *Pneumocystis murina* are robust in healthy mice but largely absent in CD40 ligand-deficient mice. J. Leukoc. Biol..

[26] Ripamonti C., Bishop L.R., Kovacs J.A. (2017). Pulmonary Interleukin-17-Positive Lymphocytes Increase during *Pneumocystis murina* Infection but Are Not Required for Clearance of *Pneumocystis*. Infect. Immun..

[27] Gherardin N.A., Souter M.N., Koay H.-F., Mangas K.M., Seemann T., Stinear T.P., Eckle S.B., Berzins S.P., d’Udekem Y., Konstantinov I.E. (2018). Human blood MAIT cell subsets defined using MR1 tetramers. Immunol. Cell Biol..

[28] Le Bourhis L., Martin E., Peguillet I., Guihot A., Froux N., Core M., Levy E., Dusseaux M., Meyssonnier V., Premel V. (2010). Antimicrobial activity of mucosal-associated invariant T cells. Nat. Immunol..

[29] Beard C.B., Fox M.R., Lawrence G.G., Guarner J., Hanzlick R.L., Huang L., del Rio C., Rimland D., Duchin J.S., Colley D.G. (2005). Genetic differences in *Pneumocystis* isolates recovered from immunocompetent infants and from adults with AIDS: Epidemiological Implications. J. Infect. Dis..

